# Impact of migrants on communicable diseases in Thailand

**DOI:** 10.1186/s12889-024-19503-9

**Published:** 2024-07-29

**Authors:** Attasuda Lerskullawat, Thitima Puttitanun

**Affiliations:** Department of Economics, Faculty of Economics, Kasetsart University, 50 Ngam Wong Wan Rd., Lat Yao, Chatuchak, Bangkok, 10900 Thailand

**Keywords:** Migrants, Health issues, Physical health, Communicable diseases, Thailand, J61, I10

## Abstract

**Background:**

While foreign migrants contribute to economic development, they may impact public health by transmitting communicable diseases to the local population. With its geopolitical position, Thailand has been a primary destination for migrants from neighbouring countries in Southeast Asia and beyond. This positioning makes it a focal point for examining the complexities of migration dynamics and its implications for public health. Through a quantitative analysis, this paper investigates the influence of foreign migrants on physical health issues in Thailand, exploring their impact on various types of communicable diseases. The utilization of provincial-level data from Thailand offers insights into the localized effects of migrant populations on public health within the country. These insights can serve as a valuable resource for researchers and policymakers who conduct comparative analyses, facilitating a deeper understanding of the complex relationship between international migration and public health worldwide.

**Methods:**

A spatial panel autoregressive model (SAR) is applied on the provincial level communicable diseases and socio-economic data in Thailand from the period 2016 to 2021.

**Results:**

The results indicate that the influence of foreign migrants on communicable diseases in Thailand varies depending on the type of disease. While an increase in migrants correlates with a higher prevalence of respiratory and other communicable diseases, it conversely reduces the prevalence of vaccine-preventable diseases. Additionally, we found that migrants do not significantly impact the prevalence of food- and water-borne diseases, insect-borne diseases, animal-borne diseases, or sexually transmitted diseases in Thailand. Additionally, other factors, such as GPP per capita, unemployment, poverty, and technology access, strongly correlate with most types of communicable diseases.

**Conclusion:**

As revealed by this study, the increase in migrants leads to a rise in respiratory and other communicable diseases, as well as a decrease in vaccine-preventable diseases, which carries significant policy implications. These results urge policymakers, the Ministry of Labour, and the Ministry of Public Health to implement tailored policies and measures to enhance public health and effectively mitigate the risk of communicable diseases transmitted by migrants in the future.

## Introduction

International migration undoubtedly impacts the economic growth of the recipient countries, primarily through the expansion of the workforce, thus contributing to an increase in GDP. A survey by the Thailand Development Research Institute (TDRI) in 2016 estimated that foreign migrants contributed 0.16% to Thailand’s GDP [1]. However, beyond its economic implications, international migration also exerts a notable influence on the health aspect of migrant sources, transit, and recipient countries. The movement of populations between locations with different health conditions can foster health risks that affect both migrants and the population in the recipient countries [2]. This is primarily attributed to the potential transmission of communicable diseases, such as respiratory illnesses, viral and parasitic infections, contagious skin diseases, and intestinal infections, from foreign workers to the local population. Additionally, the unsanitary living conditions and poor quality of life of foreign workers may exacerbate public health concerns in the receiving country, leading to an increased risk of disease transmission [2–4].

With its unique geopolitical position, Thailand serves as a primary destination for migrants from neighbouring countries in Southeast Asia and beyond. In 2020, it had the highest stock of foreign workers in Association of Southeast Asian Nations (ASEAN) [5]. This positioning makes it an ideal focal point for examining the complexities of migration dynamics and its implications for public health. Furthermore, grappling with a labour shortage and offering relatively higher wage rates compared to neighbouring nations, Thailand continues to attract a growing number of workers, particularly from the CLMV region (Cambodia, Laos, Myanmar, and Vietnam).

**Fig. 1:**
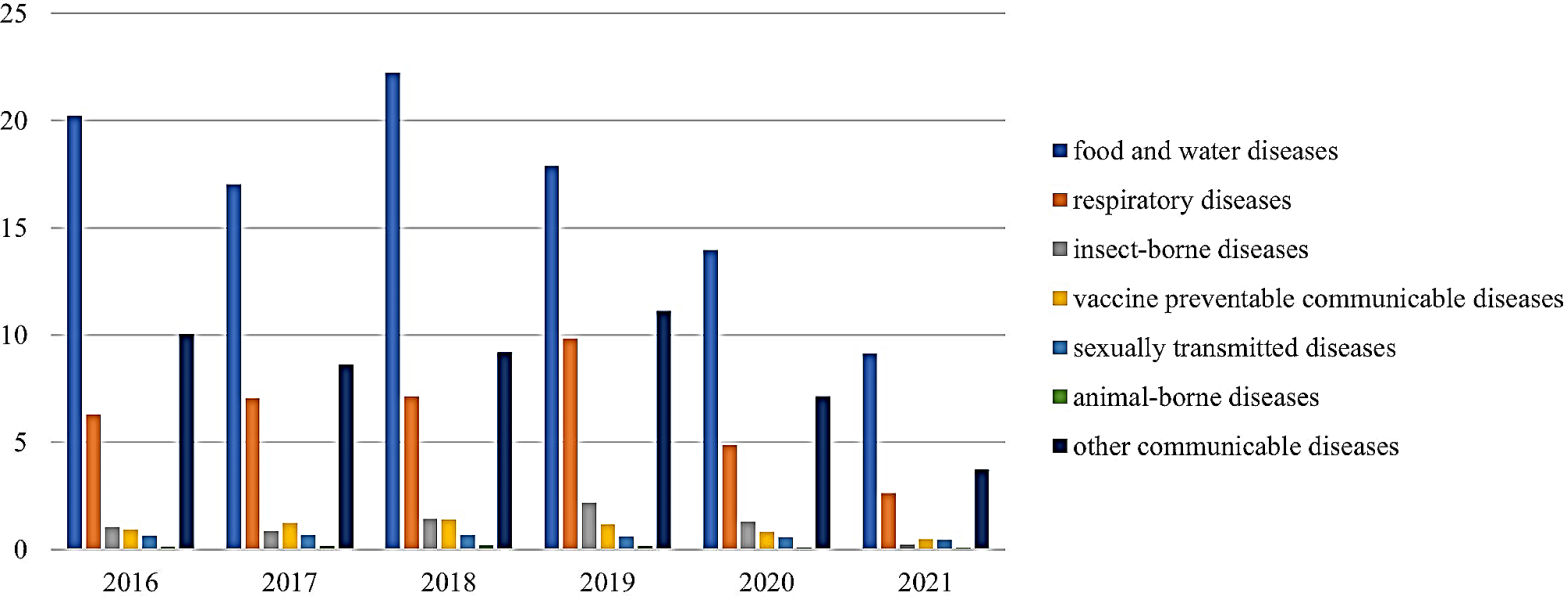
Number of patients suffering from different communicable diseases per 1000 people from 2016–2021. Source: [6]

Figure 1 shows the incidence rate of different types of communicable diseases per 1,000 people in Thailand from 2016 to 2021, indicating that those related to food and water represented the highest incidence rate, averaging 16.74 cases per 1,000 people during the period. This is followed by other communicable diseases, respiratory diseases, insect-borne diseases, vaccine-preventable communicable diseases, sexually transmitted diseases, and animal-borne diseases, which affected on average of 8.32, 6.30, 1.17, 1.00, 0.60 and 0.14 cases per 1,000 people, respectively.

**Fig. 2:**
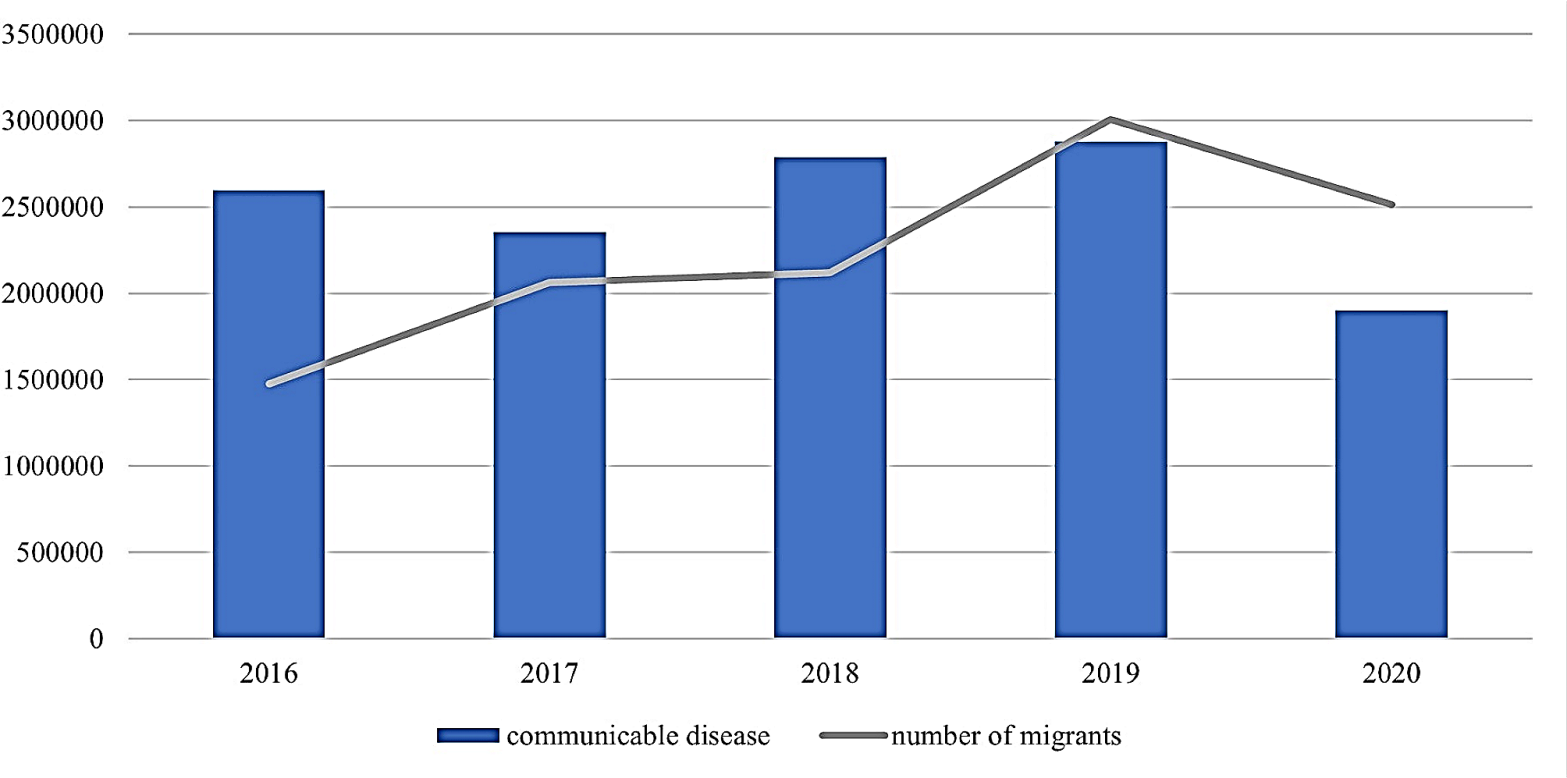
Number of migrants and patients with communicable diseases in Thailand from 2016–2021. Source: [6, 7]

Figure 2 plots the number of foreign migrants along with number of communicable diseases patients in Thailand from 2016 to 2021. It appears that the two statistics tend to move in the same direction. In 2016, there were 1,476,841 foreign migrants in Thailand, which increased to 3,005,376 in 2019. During the same period, the number of communicable disease cases also rose, from 2,593,985 in 2016 to 2,879,287 in 2019. Moreover, both figures fell in 2021, to 2,350,677 migrants and 1,103,662 communicable disease cases during the Covid-19 pandemic in 2020–2021, when travel restrictions were enforced, resulting in a reduction in both foreign migrants in the country and the spread of communicable diseases.

Therefore, the statistics shown above seems to suggest that foreign migrants can significantly impact the physical health of receiving countries through the transmission of communicable diseases, a matter of considerable interest for understanding the relationship between international migrant arrivals and health outcomes. While prior research has explored this relationship in various regions like Europe, the U.S., South America, and Asia [8–14]. Studies specific to Thailand are limited and often consist of survey or descriptive studies in localized areas [15–19].

Moreover, existing studies have typically focused on a limited set of diseases such as syphilis, respiratory diseases, malaria, and hepatitis B, rather than examining a comprehensive range. Therefore, this study aims to address these gaps by investigating the impact of foreign migrants on various types of communicable diseases in Thailand. Utilizing provincial-level data covering the period from 2016 to 2021 across all 77 provinces, this study offers a comprehensive overview of the overall impact of foreign migrants on the health of the Thai population. The findings are expected to inform public health officials and policymakers in formulating effective interventions and policies nationwide. Furthermore, addressing this issue can help dispel any misconceptions or stereotypes regarding migrants, fostering social harmony and integration between international migrant communities and the local population.

This study aims to address the gap in the literature by examining the effects of foreign migrants on various communicable diseases in Thailand, including those related to food and water, respiratory diseases, insect-borne diseases, vaccine-preventable diseases, sexually transmitted diseases, and animal-borne diseases. Using provincial data spanning from 2016 to 2021 across all 77 provinces in Thailand, this study provides comprehensive insights of the impact of foreign migrants on the health of the Thai population. The findings may be useful for public health officials and policymakers in designing appropriate interventions and policies to address this issue nationwide.

## Background on migrants in Thailand

**Fig. 3:**
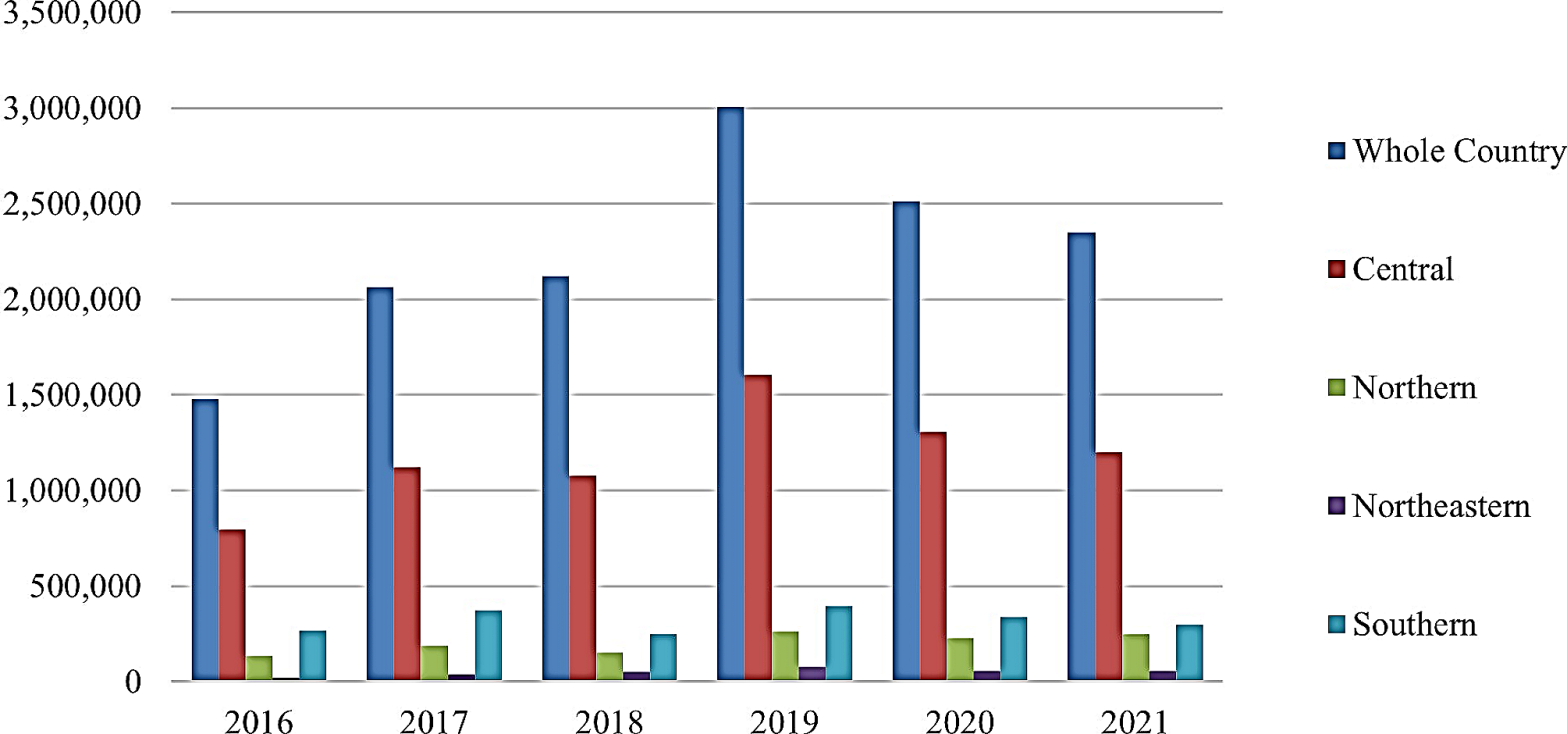
Number of migrants in Thailand from 2016–2021. Source: [7]

The Thai government has continuously revised the Thai Alien Working Act since its inception in 1978 to create official allowances, working conditions and work permits for foreign workers. Amendments include allowing workers from neighbouring countries including Myanmar, Laos, and Cambodia to work in Thailand in 1992, and implementing foreign worker management policies, including the requirement for them and their families to register with the Ministry of Interior and obtain work permits from the Ministry of Labour in 2004. Further updates, such as fee reductions and streamlining work permit processes, were introduced after 2008. Recent developments include the introduction of the Royal Enactment for Foreign Migrant Management, which developed the work permit system for foreign migrants, particularly for CLMV citizens, and addressed the management of business employing foreign migrants, along with their roles and responsibilities during 2017–2018. Additionally, the Thai Alien Working Act of 2019 was implemented to issue work permits for skilled workers and business personnel. Overtime, the Thai Alien Working Act has been updated in response to changes in economic conditions, such as the COVID-19 pandemic, and labour market conditions, including new regulations and policies for work permits, fees, and security [20, 21]. Despite these measures, labour immigration has significantly increased, rising from 1,476,841 in 2016 to 2,350,677 in 2021, representing a 59.17% increase as shown in Fig. 3. Although there was a drop in the number of foreign migrants from 2019 to 2021 due to the restriction of travel during the COVID-19 pandemic, the number still shows and upward trend from 2016.

The Central region has the highest migrant concentration accounted for 52.54% of the total number of migrants in the country, followed by the Southern (14.21%), Northern (8.95%), and Northeastern (2.26%) regions. The increase in migrants is evident across all regions with the Northeastern region experiencing the highest surge since 2016 (with 153.02% increase). Other regions with notable migrant concentrations include tourist destinations and industrial centers.

Currently, there are four main categories of foreign migrants in the country:

1. Skilled-labour migrants, including Ordinary skilled-labour type (regulated under Act No. 59) such as specialists, investors, craftsmen, and manufacturers, as well as migrants under The Thailand Board of Investment (BOI) agreement (Act No. 62).
2. Whole Life migrants (regulated under Act No. 59), who received a whole life work permit in Thailand under the Announcement of the Revolutionary Council No. 322 on 13 December 1972.
3. Minority migrants (regulated under Act No. 63/1), who do not hold Thai citizenship and have documents issued by The Ministry of Interior while awaiting work permit application approval.
4. Other Ordinary migrants, who are not considered skilled-workers and include migrants permitted to work under Memorandum of Understanding (MoU) agreements (Act No. 59 with MoU). This category encompasses individuals from CLMV. Additionally, it includes migrants from Myanmar, Laos, and Cambodia whose work permits have expired, seasonal attendees entering the country using a border pass (Act No. 63/2 issued since 13 July 2021), and migrants from Myanmar, Laos, and Cambodia who received work permits under government agreements on 20 August 2019 and 29 December 2020 [21].

**Fig. 4:**
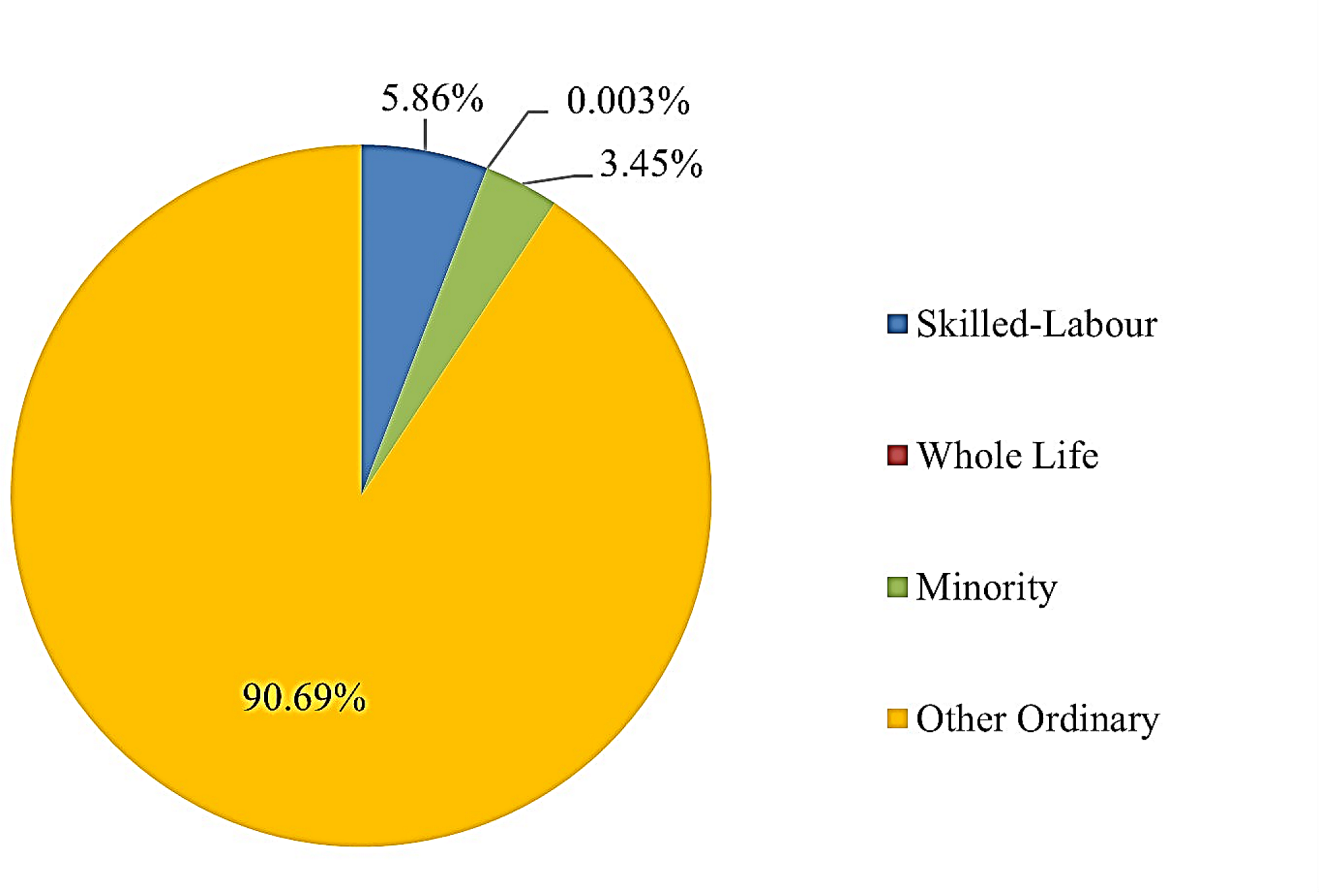
proportion of the 4 categories of migrants in Thailand in 2021. Source: [21]

Figure 4 illustrates the distribution of the four categories of migrants in Thailand in 2021. The data reveals that Other Ordinary migrants, particularly those from Myanmar, Laos, and Cambodia comprise the largest proportion, totalling 2,131,751 individuals, representing a 90.68% of the total migrant population in the country. This followed by 137,710 Ordinary skilled-labour migrants, accounting for 5.86%, 81,148 Minority migrants at 3.45%, and 68 Whole Life migrants, constituting 0.003%.

According to the Labour Market Information Administration Division, migrants in Thailand are predominantly employed in five key industries: the production sector, especially in the food and beverage sector; the construction sector; the wholesale and retail sector; the agricultural and forestry sector; and the social and service sectors. These sectors account for 19.98%, 18.98%, 16.95%, 11.71%, and 8.36%, respectively, of the total migrant population in Thailand in 2021 [22]. For migrants from the CLMV, most Cambodian migrants are employed in the construction sector, followed by the service sector and agricultural and forestry sector. Meanwhile, migrants from Laos predominantly work in the food and beverage sector, followed by the service sector and agricultural and forestry sector. In the case of migrants from Myanmar, their primary employment is in the construction sector, followed by the food and beverage sector, other production sectors, and the service sector. Finally, Vietnamese migrants mainly work in the food and beverage sector, followed by other production sectors and the service sector [23].

Given the high representation of migrants in the production and construction sectors, particularly those categorized as Other Ordinary migrants, including citizens from CLMV, who enter under MoU and government agreements, many reside in work camps and migrant dormitories provided by their employers. These accommodations can be characterized as crowed, and poorly ventilated. There is commonly little personal space. Migrant workers who have family members with them will typically rent houses near the workplace. Typically, workers reside in communities primarily composed of individuals of the same nationality. Those employed in the agricultural and service sectors often reside in accommodation provided by their employers, or the employer of the plantation allows migrants to build their own houses in an area of the plantation. Usually, this means rough housing, shared bathrooms, and pose health risks to all inhabitants due to limited access to clean water, in contrast to year-round workers, who tend to be accommodated in more robust constructions that offer decent sanitation facilities and access to clean water. During work hours, both Thai and foreign migrant workers commonly work together in the industries. Additionally, migrants establish social connections with local residents outside of work, such as at local markets or through service sector providers [21, 22, 24, 25].

Regarding health security and welfare for migrant workers in Thailand, foreign migrants, typically falling under the Other Ordinary group of migrants, can access healthcare services through the Thai social welfare system if they are registered as firm employees, or through health insurance services for migrants if they are not registered, such as household service workers, and those working in agricultural sector. According to the Thai social welfare system (Social Security Scheme), foreign migrants who are registered as firm employees must pay 5% of their income to the Social Security Fund, with employers and the government contributing 5% and 2.75% of the workers’ income, respectively. This allows them to access healthcare services offered by this scheme [26]. Foreign migrants who are not enrolled in the Social Security Scheme can access healthcare services by enrolling in the Thai health insurance service of the Ministry of Public Health, where they are required to pay yearly health check and health security insurance fees to receive healthcare services at registered hospitals. Despite the right of all migrant workers in Thailand to access these health services and employers generally covering insurance fees as a welfare benefit for workers, approximately 13% of total migrants in the country have not applied for the health insurance system [27]. Additionally, employers are sometimes reluctant to support their employees with these welfare benefits [25, 28]. This is attributed to limited access in some provinces, particularly those near the border, financial burdens for migrant workers, shortages of healthcare personnel, and inadequate funding to support migrant health insurance services [29–31]. Consequently, there remains a risk of lower healthcare access and systems for migrants in the country, posing potential health risks for the migrants and the local population, particularly concerning the transmission of communicable diseases.

## Literature review

Migrants can play a role in the transmission of communicable diseases in destination countries. This potential risk of infection arises from the migration of labourers who may carry diseases from their countries of origin. These diseases may include respiratory illnesses, insect-borne diseases, sexually transmitted diseases, food and waterborne diseases, and animal-borne diseases [4, 19]. Additionally, foreign migrant workers often face challenges such as low standards of living, poor working conditions, and limited access to public health services compared to local residents. This disparity can increase the risk of transmitting communicable diseases to people in destination countries, such as respiratory diseases, intestinal infections, disease caused by viruses and bacteria, skin infections, and animal-borne diseases [2, 3, 32–34]. Some studies have found that migrants can place a strain on the healthcare system, as they may have higher rates of certain health conditions and may be more likely to use emergency services. This can result in longer wait times and reduced access to care for the local population [20, 35].

Ibánez et al. [13] investigated the impact of migrants on communicable diseases in Colombia and found associations with an increase in vaccine-preventable diseases such as tuberculosis and chickenpox, as well as sexually transmitted diseases such as AIDS and syphilis. The study also found that older age groups faced a higher risk of sexually transmitted diseases in the destination country. Lifshits and Neklyudova [12] observed an increase in communicable diseases among the population in Rusia, including syphilis, hepatitis B and hepatitis C, which they linked to various factors such as poverty, unemployment, and drug addiction. Survey studies by Vonneilich et al. [14] and Green et al. [9] in 28 European countries and England suggested that immigrants might be associated with overall physical health problems for both the population of the destination countries and the areas where migrants have settled. Additionally, older population proportion was identified as another factor increasing physical health problems for the population. Literature surveys conducted by Castelli and Sulis [10] and Barnett and Walker [36] indicated that migrants might play a role in the spread of sexually transmitted diseases such as AIDS, vaccine-preventable diseases (such as measles, chickenpox and hepatitis B), respiratory diseases (such as pertussis and tuberculosis), and insect-borne diseases in destination countries. Similar observations were made by Rechel et al. [37] and Montiel et al. [38] in their studies of Europe and Central Asia. However, Deb and Gurevich [11] did not find a significant effect of migrants on physical health problems in Indonesia. Instead, they found that factors such as gender, age, and education level were important influences on health problems in the country.

Regarding studies conducted about Thailand, Kunnu and Pasunon [39] surveyed Surat Thani province and noted that migrants could play a role in the spread of communicable diseases in the area. They also highlighted factors such as access to healthcare services and migrants’ living conditions as influencing the prevalence of communicable diseases. Phakamach et al. [19] conducted interviews and literature surveys, suggesting that migrants might be associated with the spread of physical health problems such as AIDS and other sexually transmitted diseases. Rruenkrew [16] conducted a descriptive study and noted that Thai labour migration to Germany and Japan could result in physical health problems for both the migrant labourers and the populations of destination countries. The study highlighted a lack of healthcare services for workers, which could increase the risk of communicable diseases, particularly sexually transmitted ones. Similarly, Klanarong [15] found that Thai labour migrants to Malaysia might increase the risk of spreading sexually transmitted diseases to the Malaysian population.

Thongpan [25], Klanarong [15], and Laosai and Teeravisit [17] conducted descriptive analyses and literature surveys, indicating that foreign labour immigration could contribute to public health challenges in Thailand due to a shortage of human resources in the public health system. This shortage may increase the risk of physical health issues among Thai people. Moreover, a rise in the number of migrants could result in a higher incidence of communicable diseases such as malaria and tuberculosis among the native population. These studies emphasize the importance of robust healthcare services in addressing communicable diseases in the country.

Apart from the effect of migrants on the communicable diseases of the local population, other factors also influence the prevalence of communicable diseases in the country. Personal behaviours such as non-hygienic food consumption, unsafe sexual practices, drug and alcohol addiction, and poor living habits significantly increase the risk of health problems and communicable diseases. Gender and age are also critical demographic factors that influence the risk of communicable diseases and health problems. For example, younger individuals are at a higher risk of vaccine-preventable diseases, males and younger populations are more susceptible to sexually transmitted diseases, and the elderly are more vulnerable to respiratory diseases [40, 41].

Social factors, including the quality of living environments, access to healthcare, and public infrastructure, play a substantial role in the transmission of communicable diseases. Poor living conditions, underdeveloped infrastructure, and a lack of clean water, fresh air, and good nutrition ca. significantly increase the transmission rates of foodborne infections, respiratory diseases, and insect and animal-borne diseases. Additionally, limited access to healthcare is a major problem contributing to the spread of communicable diseases and general health issues in many countries [42, 43]. Insufficient education and technological infrastructure limit individuals’ knowledge about health information and awareness, reducing their ability to access necessary goods and services, income sources, and health services. This situation increases the risk of health problems and communicable diseases [40, 42, 44–46].

Economic factors, including economic growth, income levels, unemployment, and poverty rates, also influence the risk of communicable diseases and physical health problems. Higher economic growth and income levels, along with lower poverty and unemployment rates, provide people with better access to basic human needs and improve their quality of life, which in turn enhances access to healthcare systems and services. Increased income and a better economy enable individuals to afford healthcare insurance and medical expenses, thereby reducing physical health problems and the risk of communicable diseases. Improved economic conditions also allow governments to invest more in public health services and welfare, thereby mitigating the risk of communicable diseases within the country [40, 43, 47, 48].

## Methods

Secondary data were collected from the 77 provinces of Thailand covering the period 2016 to 2021. Information on communicable diseases was obtained from the ThaiHealthStat website, which collects data on the health conditions and diseases of the Thai population from the Department of Disease Control. Data on the number of foreign migrants, and on the economic and social factors used as control variables, namely gross provincial product, the unemployment rate, education, healthcare services, technological infrastructure, health behaviour risk, gender, and age of the population, were collected from Thai National Statistics. Summary of the data used in this study is shown in Table 1 and is available from the corresponding author upon request.

**Table 1:** Variables used in the study

Variable	Symbol	Calculation detail
Migrants per capita	migrant	Proportion of migrants to population in the province
Communicable diseases		
Patients with communicable diseases per capita	phyhealth	Proportion of patients with communicable diseases to population in the province
Patients with food- and water-borne diseases per capita	food	Proportion of patients with food- and water-borne diseases to population in the province
Patients with respiratory diseases per capita	respir	Proportion of patients with respiratory diseases to population in the province
Patients with insect-borne diseases per capita	insect	Proportion of patients with insect-borne diseases to population in the province
Patients with vaccine-preventable communicable diseases per capita	vac	Proportion of patients with vaccine-preventable communicable diseases to population in the province
Patients with sexually transmitted disease per capita	sexual	Proportion of patients with sexually transmitted diseases to population in the province
Patients with animal-borne diseases per capita	animal	Proportion of patients with animal-borne diseases to population in the province
Patients with other communicable diseases per capita	others	Proportion of patients with other communicable diseases, including haemorrhagic conjunctivitis, hand-foot-and mouth Disease, melioidosis, scarlet fever and fevers of unknown origin, to population in the province
Control variables		
Economic condition	GPP	Real gross provincial product per capita
Unemployment	unem	Proportion of unemployed person to population in the province
Poverty	pov	Proportion of poor people to population in the province
Education	edu	Average years of education of the population in the province
Health services	doc	Proportion of number of doctors to population in the province
Technological infrastructure	tech	Proportion of number of computers to population in the province
Risky health behavior	drug	Proportion of drug crime cases in the province to population in the province
Gender	male	Proportion of males to population in the province
Old population	old	Proportion of population over the age of 60 to population in the province

To investigate the effect of foreign migrants on different communicable diseases, including ones related to food and water, and respiratory, insect-borne, vaccine-preventable, sexually transmitted, and animal-borne diseases, and other communicable diseases in Thailand, this study employed the following the model specification^1^, as used by Ibanez et al. [13], Lifshits and Neklyudva [12]:

1 $$\begin{array}{l}\:{Y}_{i,t}=\:{\alpha\:}_{i}+{{\beta\:}_{1}migrant}_{i,t}+{\beta\:}_{2}{GPP}_{i,t}+{\beta\:}_{3}{unem}_{i,t}\\+\:{\beta\:}_{4}{pov}_{i,t}+\:{{\beta\:}_{5}edu}_{i,t}+{{\beta\:}_{6}doc}_{i,t}{{+{{\beta\:}_{7}tech}_{i,t}+\beta\:}_{8}drug}_{i,t}\\+{{\beta\:}_{9}male}_{i,t}+{{\beta\:}_{10}old}_{i,t}\:+{\epsilon\:}_{i,t}\end{array}$$

where α_i_ is the individual province’s specific effect, using the control variables that might influence the prevalence of communicable diseases in the local population.

*Y*_*i, t*_ is the proportion of local population patient^2^ with different physical health problems including:

phyhealth_i, t_, the proportion of all patients with communicable diseases to the population in province *i* in year *t*,

food_i, t_, the proportion of patients with food- and water-borne diseases to the population in province *i* in year *t*. Such diseases include diarrhea and food poisoning.

respir_i, t_, the proportion of patients with respiratory diseases to the population in province *i* in year *t*. Respiratory diseases include influenza and pneumonia.

insect_i, t_, the proportion of patients with insect-borne diseases to the population in province *i* in year *t*. Such diseases include dengue virus, malaria and Chikungunya virus.

vac_i, t_, the proportion of patients with vaccine-preventable communicable diseases to the population in province *i* in year *t*. Such diseases include tuberculosis, hepatitis B, measles, mumps and chickenpox.

sexual_i, t,_ the proportion of patients with sexually transmitted diseases to the population in province *i* in year *t*. These include syphilis and gonorrhea.

animal_i, t_, the proportion of patients with animal-borne diseases to the population in province *i* in year *t*, including those with leptospirosis and scrub typhus.

others_i, t_, the proportion of patients with other communicable diseases, including hemorrhagic conjunctivitis, hand-foot-and mouth disease, melioidosis^3^, scarlet fever and fevers of unknown origin, to the population in province *i* in year *t.*

migrant_i, t_, the proportion of foreign workers to the population in province *i* in year *t*. According to previous research, an increase in the number of foreign migrants can lead to an increase in the number of patients with communicable diseases because of their lower living and working conditions. Migrants might also carry infectious diseases from their home country. In addition, labour immigration can create public health problems due to the lack of public health and health insurance system funding to cope with the issue, creating more physical health risk in the destination population.

The economic and social control variables were as follows:

GPP_i, t_, real gross provincial product per capita, used to represent economic conditions in province *i* in year *t*. Better conditions can lead to improved economic opportunities, which in turn result in a better standard of living, better health, and a reduction in the risk of contracting communicable diseases. However, an increase in GPP per capita might also signify an increase in economic activities. With such an increase, there will be more social interaction and movement between people, which could lead to an increase in the spread of communicable diseases.

unem_i, t_, the proportion of unemployed people to the population in province *i* in year *t*. This variable is another control for economic opportunities. A high unemployment rate signifies lower economic opportunities and public health access due to lower income, thus increasing the risk of contracting communicable diseases. It could also indicate weaker economic activity and less social interaction, which will reduce the spread of communicable diseases.

pov_i, t_, the proportion of the population under the poverty line to the overall population in province *i* in year *t*. A high level signifies poorer economic opportunities, a lower quality of life, and a higher risk of contracting communicable diseases.

edu_i, t_, the average number of years of education of the population in province *i* in year *t*. A higher education level can provide better economic opportunities and give people more knowledge of how to take care of themselves and avoid the risks of contracting communicable diseases.

doc_i, t_, the proportion of the number of doctors to the population in province *i* in year *t*. An increase in this proportion may suggest that the province has a better public health service with more healthcare personnel, which can help prevent the population from contracting diseases.

tech_i, t_, the proportion of the number of computers to the population in province *i* in year *t*, showing the technological infrastructure of the province. An increase in this proportion could show a higher level of access to technology, which can lead to a better quality of life, greater access to health information, knowledge of health risk prevention, therefore lowering the risk of contracting diseases.

drug_i, t_, the proportion of drug-related crime cases to the population in province *i* in year *t*. An increase in this proportion shows a higher risk of being exposed to drugs and illegal substances, which in turn can lead to poor nutrition and hygiene, weakening the immune system, worsening health conditions, and increasing the risk of contracting diseases.

male_i, t ,_ the proportion of the male population to the overall population in province *i* in year *t*. This ratio can discern which gender is more susceptible to different types of diseases.

old_i, t_, the proportion of older people (over 60) to the population in province *i* in year *t*. The ratio can discern whether older people are more susceptible to different types of diseases.

$\:{\epsilon\:}_{i,t}$ is an error term.

The number of patients with communicable diseases in each province can be geographically influenced by neighbouring observations, known as spatial correlation, which refers to the tendency of observations in close proximity to each other to be more similar than ones that are farther apart. Outbreaks of communicable diseases in one area can spread to neighbouring areas through various means, such as travel, transportation, and human contact. These factors may consequently affect the health of individuals in the neighbouring areas, leading to spatial autocorrelation. Spatial regression techniques allow researchers to account for spatial dependencies in the data. Previous studies have employed spatial regression to explore the spatial distribution of diseases such as tuberculosis [50–52], HIV/AIDS [53–55], dengue [56, 57], and other communicable diseases in relation to migrant populations [58, 59].

The spatial panel autoregressive model (SAR) combines elements of panel data analysis, spatial dependence, and autoregressive modelling as follow:

- Panel data: The data set in the study is 77 provincial level data over multiple time periods (2016–2021). This type of data allows for both cross-sectional and time-series analysis, providing insights into both individual province and temporal variations. Each province might have provincial-specific factors that remain constant over time such as proximity to the bordering countries, average humidity, etc. that can relate to the prevalence of diseases. There may be unobserved characteristics that affect both the independent and dependent variables such as culture, lifestyles and eating habits that can also affect the prevalence of diseases. Therefore, panel data method controlling for provincial fixed effect is needed.
- Autoregressive: Observations within the same province might be correlated over time due to temporal dynamics. The SAR model takes into account that the current value of a variable can be influenced by its past values.
- Spatial dependence: Spatial dependence occurs when observations in one location are correlated with observations in nearby locations. As in the case of diseases, the neighbouring provinces might be affected if there is an outbreak in the nearby locations.

Therefore, this study uses the spatial panel autoregressive model to provide more accurate estimates of the relationship between migrants and the proportion of patients with communicable diseases. The weight matrix (ρW_ij_ y_j_) is included in the model to account for the effect of spatial correlation from province *j* (y_j_), which could be transmitted to nearby province *i*. ρ is the coefficient of W, showing the effect of spatial correlation from province *j* which can be transmitted to nearby province *i*, and W_ij_ is a weight matrix variable, which has a value of 1 if province j is near to province *i*, and 0 otherwise. Fixed-and random-effects spatial panel autoregressive models are considered, based on the Hausman test.

A summary of the statistics relating to the variables is shown in Table 2 and a correlation matrix is shown in Table 3. As we can see from Table 3, none of the correlation coefficients exceed the commonly accepted threshold, a magnitude of 0.8 [60], indicating the absence of significant multicollinearity among the independent variables.

**Table 2:** Summary statistics

Variable	Observations	Mean	Standard Deviation	Minimum	Maximum
phyhealth	462	0.035	0.020	0.004	0.323
food	462	0.018	0.015	0.001	0.293
respire	462	0.006	0.003	0.0003	0.020
insect	462	0.001	0.001	5.89e-06	0.007
vac	462	0.001	0.0006	0.0001	0.005
sexual	462	0.0008	0.0005	0.000	0.002
animal	462	0.0002	0.0003	0.000	0.003
others	462	0.008	0.006	0.0001	0.037
migrant	462	0.032	0.055	0.0002	0.409
**Control Variable**					
GPP	462	0.118	0.145	0.024	0.734
unem	462	0.007	0.006	0.000	0.075
pov	462	9.377	8.118	0.000	46.540
edu	462	8.248	0.933	5.560	11.260
tech	462	0.062	0.038	0.009	0.252
doc	462	0.0004	0.0002	0.00005	0.002
drug	462	0.003	0.002	0.000	0.012
male	462	0.484	0.017	0.403	0.504
old	462	0.166	0.032	0.098	0.255

**Table 3:** Correlation Matrix

	migrant	gppcap	unem	povrate	edu	tech	doc	drug	male	age
migrant	1.00									
gppcap	0.58	1.00								
unem	0.16	0.24	1.00							
povrate	-0.31	-0.44	-0.04	1.00						
edu	0.38	0.60	0.34	-0.46	1.00					
tech	0.43	0.57	0.36	-0.40	0.65	1.00				
doc	0.46	0.57	0.23	-0.40	0.59	0.63	1.00			
drug	0.002	-0.08	-0.06	0.05	-0.03	-0.21	-0.08	1.00		
male	-0.48	-0.27	-0.09	-0.03	-0.13	-0.37	-0.34	0.18	1.00	
age	-0.17	-0.14	-0.08	-0.24	0.11	0.10	0.12	-0.31	0.10	1.00

## Findings and discussion

As can be seen in Table 4, while the overall impact of foreign migrants on communicable disease contraction in the local population was found to be insignificant (column 1), an intriguing finding was revealed, in that the effect of foreign migrants on different types of communicable diseases varied significantly. To be specific, food- and water-borne diseases, insect-borne diseases, sexually transmitted diseases, and animal-borne diseases were not affected by the number of migrants in the area (columns 2, 4, 6, and 7). However, respiratory communicable diseases (column 3), namely influenza and pneumonia, as well as other communicable diseases (column 8), hemorrhagic conjunctivitis, hand-foot-and mouth disease, melioidosis, scarlet fever and fevers of unknown origin, were positively related to the proportion of foreign migrants in the area. This might be because the poor living and working conditions and hygiene of most of the foreign migrant workers in Thailand increase exposure to contaminated environments, which can lead to bacterial infection such as influenza and melioidosis. Moreover, an increase in the proportion of foreign migrants can result in a higher transmission of viruses through the air, nasal discharge, saliva, and skin-to-skin contact, as well as water contact, thereby increasing the risk of contracting other communicable diseases. This result is similar to those obtained in previous studies [2, 3, 33, 61–63].

Interestingly, an increase in the foreign migrants per population leads to a reduction in the contraction of vaccine-preventable communicable diseases amongst the local population (column 5). This might be due to the Thai government’s control plan of 2017–2021, which aimed to prevent such diseases. The plan involved developing a registration system for migrants and implementing policies to monitor and control the spread of diseases among foreign workers entering the country, particularly in provincial areas [64]. Therefore, migrants in Thailand might be immune to such vaccine-preventable diseases, thus helping to reduce their overall prevalence.

Regarding control variables, the economic indicator GPP per capita is positively correlated to all types of communicable diseases (columns 1–6 and 8) except for animal-borne ones (column 7). A rise in the indicator may signify an increase in economic activities among the population of an area, which can lead to more social interaction and movement, and which in turn can increase the spread of communicable diseases. Conversely, an inverse relationship can be observed between unemployment and economic activity; an increase in unemployment suggests a decrease in economic activity, hence reducing the spread of communicable diseases (columns 5–8). In addition, a rise in the proportion of the population living in poverty increases the prevalence of communicable diseases (columns 5 and 7). Being in poverty can lead to limited access to vaccines and healthcare, thereby increasing the risk of contracting vaccine- preventable diseases.

In relation to social factors, the number of doctors per population, a proxy for improved healthcare services, has a negative effect on insect-borne diseases (column 4). This suggests that improving the public health infrastructure can be an effective means of preventing the spread of such diseases. An increase in the use of technological devices, representing development of the country’s infrastructure, helps reduce the prevalence of most communicable diseases, apart from animal-borne ones (columns 1–6 and 8). This might be because with improved communication, people can communicate more effectively and share information about diseases, preventive measures, and treatments, which can help reduce their spread. The drug crime cases to the population variable is positively related to vaccine- preventable communicable diseases (column 5). This finding suggests that a higher risk of exposure to drugs and illegal substances can lead to poor nutrition and hygiene, weakening the immune system, worsening health conditions, and therefore increasing the risk of contracting such diseases.

Regarding the population structure factors, the proportion of the male population in a province is shown to be positive in animal-borne diseases and other communicable diseases (columns 7–8). This suggests that males might be more susceptible to contracting these types of disease than women. The proportion of older people in a province is shown to be consistently negative in relation to almost all types of communicable diseases (columns 1–3, 5–6 and 8). Due to their fragility and susceptibility to contracting diseases, older citizens tend to be more cautious and less socially active, which may result in a reduction in the prevalence of communicable diseases in their area.

Finally, the significance of the weight matrix (W) coefficient in all regressions shown in Table 4 suggests that geographical factors have an influence on the proportion of communicable disease patients in each province. Therefore, it is appropriate to use the specific effect spatial autoregressive model.

**Table 4:** Prevalence of Communicable diseases

Variable	phyhealth	food	Respir	insect	vac	sexual	animal	others
	(1)	(2)	(3)	(4)	(5)	(6)	(7)	(8)
Migrant	**0.0004**	**-0.031**	0.01**	**0.0006**	-0.001*	**-0.0004**	**0.0001**	0.016**
	**(0.040)**	**(0.037)**	**(0.005)**	**(0.002)**	**(0.0008)**	**(0.0006)**	**(0.0004)**	**(0.007)**
GPP	0.129***	0.073*	0.022***	0.006**	0.001***	0.001***	0.0006	0.016*
	(0.048)	(0.044)	(0.006)	(0.003)	(0.0004)	(0.0004)	(0.0004)	(0.009)
unem	-0.184	-0.098	-0.0004	0.002	-0.006*	-0.006***	-0.004***	-0.052*
	(0.156)	(0.144)	(0.019)	(0.009)	(0.004)	(0.002)	(0.001)	(0.028)
pov	-0.00009	-0.0001	-2.5e-05	2.53e-06	7.13e-06*	-8.63e-07	8.26e-06***	5.31e-05
	(0.0001)	(0.0002)	(2.25e-05)	(1.04e-05)	(4.13e-06)	(2.91e-06)	(1.66e-06)	(3.31e-05)
edu	-0.0004	-0.001	-3.33e-05	-1.15e-05	-5.26e-05	-4.66e-05	8.10e-06	0.0002
	(0.002)	(0.002)	(0.0003)	(0.0001)	(4.65e-05)	(3.56e-05)	(2.18e-05)	(0.0004)
doc	6.465	3.333	-0.457	-0.921**	0.174	0.271	0.197	2.277
	(10.942)	(10.093)	(1.322)	(0.410)	(0.190)	(0.225)	(0.133)	(1.930)
tech	-0.174***	-0.066*	-0.034***	-0.010***	-0.002***	-0.002***	-0.0003	-0.029***
	(0.044)	(0.038)	(0.006)	(0.002)	(0.001)	(0.0006)	(0.0004)	(0.007)
drug	-0.441	-0.513	0.057	-0.008	0.036***	-0.016	0.003	0.004
	(0.506)	(0.468)	(0.060)	(0.028)	(0.012)	(0.010)	(0.004)	(0.089)
male	0.214	-0.115	-0.039	0.018	-0.003	-0.004	0.028***	0.253***
	(0.534)	(0.492)	(0.063)	(0.029)	(0.003)	(0.003)	(0.005)	(0.095)
old	-0.484***	-0.299***	-0.058***	-0.006	-0.003**	-0.004**	-0.0008	-0.080***
	(0.075)	(0.062)	(0.009)	(0.003)	(0.001)	(0.0009)	(0.0006)	(0.013)
W	0.239***	0.125*	0.453***	0.629***	0.563***	0.157**	0.266***	0.411***
	(0.066)	(0.074)	(0.044)	(0.035)	(0.038)	(0.062)	(0.056)	(0.047)
Observations	462	462	462	462	462	462	462	462
Number of id	77	77	77	77	77	77	77	77
Hausman test statistic	74.75	42.12	41.98	-0.25	236.62	60.71	54.00	48.80
Model	FE	FE	FE	FE	FE	FE	FE	FE

**Notes**: Standard errors are shown in parentheses. *,** and *** signify 10%, 5% and 1% levels of significance respectively

In summary, the impact of foreign migrants on communicable diseases in Thailand varies depending on the type of disease. An increase in the proportion of foreign migrants in a province leads to a rise in the prevalence of respiratory and other communicable diseases, but a fall in that of vaccine-preventable diseases. However, the study found no evidence that migrants have a significant impact on the prevalence of food- and water-borne, insect-borne, animal-borne, or sexually transmitted diseases.^4^

## Strengths and limitations

The study contributes to the literature in four main ways. Firstly, it examines not only the overall impact of foreign migrants on communicable diseases in Thailand but also investigates their effects on different disease types. Secondly, it fills gaps in the empirical literature by providing evidence specific to Thailand. While the study is based on data from Thailand, its findings hold relevance for other developing nations facing similar challenges in managing public health amid international migration. Overall, previous research on the impact of migrants on physical health has primarily focused on developed countries, leaving a knowledge gap regarding the Thai context. Thirdly, unlike previous studies that focused on specific provinces or areas, or relied on descriptive analyses, this study employs an empirical approach using provincial-level data, offering a more comprehensive understanding of the impact across the entire country. Fourthly, this study adopted the spatial panel autoregressive model (SAR), which explicitly accounts for spatial dependencies among observations. This is crucial when analysing data in which nearby observations are likely to influence each other, as is often the case in disease transmission. Such a method provides unbiased and more efficient estimates compared to models that ignore spatial dependencies.

Nevertheless, due to the unavailability of consecutive provincial data on illegal migrants in Thailand^5^, this research may have limitations in capturing the overall impact of migrants on the physical health of the native population. Additionally, the proportion of doctors^6^ may not fully capture the accessibility of healthcare services across different population groups. Migrants may potentially encounter more challenges in accessing healthcare services compared to native population. Therefore, it is imperative to approach estimations with caution and recognize these potential limitations.

## Policy implications

Based on the findings of this study, the main policy implications are as follows: Firstly, the Department of Health and the Department of Disease Control should enhance their monitoring and control measures concerning foreign migrants, particularly focusing on respiratory illnesses and other communicable diseases. This can be achieved by collaborating with the Department of Employment and the Department of Public Welfare to improve and update the health check system for migrants and establish a tracking mechanism to effectively monitor the health status of migrants. Secondly, the government and the Department of Health must ensure that migrants have access to sufficient health welfare. Therefore, updated health insurance policies should be considered for migrants due to the challenges they often face in accessing healthcare services. Additionally, the government’s healthcare budget plans should focus on improving access to healthcare for both native and migrants equally. Furthermore, Memorandums of Understanding (MoUs) and contracts foreign migrants should include clear and accurate information about housing provisions and standards to protect the health and well-being of migrant workers. Migrant workers in Thailand often live in substandard accommodation, which can increase the risk of contracting and transmitting diseases. Therefore, the government should ensure that housing provided by employers meets minimum standards of adequate and decent living conditions.

In addition, other factors such as economic conditions have been found to strongly relate to most types of communicable diseases. Consequently, the government should strive to improve economic conditions and enhance the standard of living by reducing poverty and unemployment. This can be achieved through the Thai Economic and Social Development Plan No.13 (2023–2027), which should prioritize the development of human capital, productivity enhancement, fair income distribution, and reducing unemployment. Technology should be utilized in the healthcare system to develop preventive measures, maintain up-to-date data on migrant workers’ health and diseases, and establish effective early warning systems to address the potential spread of communicable diseases. Additionally, the population structure, including gender and age distribution, should be carefully considered when formulating future health control policies, as variations in these demographic factors may lead to different impacts on communicable diseases within the country.

## Conclusion

The study has investigated the impact of foreign migrants on different types of communicable diseases in Thailand. Utilizing provincial data from all 77 provinces in Thailand from 2016 to 2021, the findings reveal that the influence of the foreign migrants on communicable diseases in Thailand varies depending on the disease type. A rise in foreign migrants per population leads to an increase in respiratory and other communicable diseases, while reducing the prevalence of vaccine-preventable ones. There is no evidence that migrant concentration has a significant impact on the prevalence of food- and water-borne, insect-borne, animal-borne, or sexually transmitted diseases in Thailand. Therefore, solely examining the overall prevalence of communicable diseases may lead to misleading conclusions and could result in inappropriate policy development by the authorities regarding migrants.

The findings of this study can provide valuable insights for other countries facing similar challenges. By understanding the varying impact of migrants on different diseases, other nations can adapt and develop strategies to address public health concerns associated with migrants. This study’s results can guide policymakers, healthcare professionals, and researchers in making informed decisions and implementing measures to protect public health and mange communicable diseases.

## Data Availability

The datasets used in this study are available from the corresponding author on reasonable request.

## Footnotes

1 The statistical software STATA was used in our analysis.

2 We employ the patient-to-population proportion as a normalization technique to account for population size disparities across provinces, enabling equitable comparisons between provinces with different population sizes.

3 An infectious disease caused by Burkholderia pseudomallei bacteria, usually found in contaminated soil and water (see [49]).

4 As a robustness check, we used the Fixed Effect model without correcting for spatial dependence. The results are similar to those in Table 4. The effect of foreign migrants on communicable diseases varied depending on the type of diseases. Respiratory and other communicable diseases are still positively related to the number of migrants in the area. However, migrants are not statistically influence the vaccine-preventable communicable diseases in this technique. Other control variables also show similar qualitative results to those in Table 4. However, the fixed effect model only controls for provincial-specific effects without accounting for spatial dependence and temporal dynamics. Therefore, the Spatial panel autoregressive model outcomes in Table 4 would provide more accurate results.

5 The Thai government has made efforts to account for all migrants entering the country, ensuring that illegal migrants have their status changed to legal immigrants after nationality verification. Therefore, the data on migrants in the analysis should cover the majority of the foreign migrants in the country.

6 Due to the unavailability of other social welfare measures at the provincial level, number of doctors per population is used to represent public healthcare services by province each year.

## References

[1] Naewna. Foreign migrants policy: Adapting Perspectives in Thailand’s Aging Society [in Thai]. Retrieved from: https://www.naewna.com/likesara/635106. Accessed 14 June 2024. 2022

[2] Hossin MZ. International migration and health: it is time to go beyond conventional theoretical frameworks. BMJ Global Health. 2020;5:1–7. 10.1136/bmjgh-2019-001938. 10.1136/bmjgh-2019-001938 PMC7053782 32180999

[3] Virupaksha HG, Kumar A, Nirmala BP. Migration and mental health: an interface. J Nat Sci Biology Med. 2014;5(2):233–9. 10.4103/0976-9668.136141. 10.4103/0976-9668.136141 PMC4121889 25097389

[4] Shetty AK. Infectious diseases among Refugee Children. Children. 2019;6(159):1–21. 10.3390/children6120129. 10.3390/children6120129 PMC6955676 31783605

[5] Asian Development Bank Institute, International Labour Organization, and Organisation for Economic Co-operation and Development. (2022). Labor Migration in Asia: COVID-19 Impacts, Challenges, and Policy Responses. Retrieved from: https://www.adb.org/sites/default/files/publication/797536/labor-migration-asia.pdf. Accessed 11 May 2024.

[6] ThaiHealthStat. (2020). Thai Health Statistic. Retrieved from: https://www.hiso.or.th/thaihealthstat/, Accessed 14 September 2022.

[7] Thai National Statistical Office. (2022). Labour statistic. Retrieved from: http://statbbi.nso.go.th/staticreport/page/sector/th/02.aspx, Accessed 14 September 2022.

[8] Moullan Y, Jusot F. Why is the ‘healthy immigrant effect’ different between European countries? Eur J Pub Health. 2014;24:80–6. 10.1093/eurpub/cku112. 25108002 10.1093/eurpub/cku112

[9] Green MA, Subramania SV, Vickers D, Dorling D. Internal migration, area effects and health: does where you move to impact upon your health? Soc Sci Med. 2015;136–7. 10.1016/j.socscimed.2015.05.011. 10.1016/j.socscimed.2015.05.011 25978650

[10] Castelli F, Sulis G. Migration and infectious diseases. Clin Microbiol Infect. 2017;23:283–9. 10.1016/j.cmi.2017.03.012. 28336382 10.1016/j.cmi.2017.03.012

[11] Deb P, Gurevich T. (2017). The effect of internal migration on health of adults in Indonesia. U.S. International Trade Commission Economics Working Paper Series, 25-B, 1–41. Retrieved from: https://www.usitc.gov/publications/332/migration-health-wp-pdf_0.pdf. Accessed 20 August 2022.

[12] Lifshits M, Neklyudova N. (2020). Migration, Infectious Diseases and Drug Addiction in Russia. Retrieved from: https://www.medrxiv.org/content/10.1101/2020.10.09.20209791v1.full.pdf, Accessed 15 August 2022.

[13] Ibánez AM, Rozo S, Urbina MJ. Forced Migration and the spread of Infectious diseases. Inter-American Dev Bank Discussion Paper. 2020;834:1–70. 10.1016/j.jhealeco.2021.102491. 10.1016/j.jhealeco.2021.102491 34375854

[14] Vonneilich N, Bremer D, Knesebeck OVD, Lüdecke D. Health patterns among migrant and non-migrant middle and older-aged individuals in Europe-analyses based on share 2004–2017. Int J Environ Res Public Health. 2021;18:1–13. 10.3390/ijerph182212047. 10.3390/ijerph182212047 PMC8622058 34831800

[15] Klanarong N. Labour Migration of Females in four southern border provinces to Malaysia [in Thai]. Thaksin J. 2005;8(2):1–11.

[16] Rruenkrew P. Research on rights of Thai Woman in the case of Labour Immigration [in Thai]. Bangkok, Thailand: Office of the National Human Rights Commission of Thailand; 2009.

[17] Laosai S, Teeravisit A. (2012) Access to Health Services of Burmese Migrant Workers in Industrial Factories in Khon Kaen Province [in Thai], Retrieved from: https://gsbooks.gs.kku.ac.th/55/cdgrc13/files/hmp5.pdf, Accessed on 1st September 2023.

[18] Kangkarn P. The impact of Foreign Worker Trade on National Security in Economy, Society and politics: Case Study in Chaiyaphum Province [in Thai]. Ratchaphruek J. 2017;15(3):55–62.

[19] Phakamach P, Prasongsang C, Chaisanit S, Chuiypetch C, Yousukee T. The guidelines for managing Foreign workers in the Eastern Part of Thailand [in Thai]. J Humanit Social Sci Univ Phayao. 2019;7(2):190–229.

[20] Aenihon P. Foreign workers: implementation and Administration in Thailand [In Thai]. J Arts Manage. 2018;2(2):117–32.

[21] Thai Foreign Workers Administration Office. (2022). Law governing the management of alien. Accessed on: https://www.doe.go.th/prd/alien/law/param/site/152/cat/102/sub/0/pull/category/view/list-label. Accessed 10th August 2022.

[22] Labour Market Information Administration Division. (2021). Full Employment for all Ages 2022. Accessed at: https://www.doe.go.th/prd/assets/upload/files/lmia_th/969b768c91b93ebbd0474738c62c671c.pdf. Accessed 12th May 2024.

[23] Thaipublica. (2024). Foreign Workers Statistics for 15 Years in Thailand (in Thai). Retrieved from: https://thaipublica.org/2024/04/foreign-worker-statistics-for-15-years-in-thailand/#:~:text=%E0%B8%82%E0%B9%89%E0%B8%AD%E0%B8%A1%E0%B8%B9%E0%B8%A5%E0%B8%88%E0%B8%B2%E0%B8%81%E0%B8%AA%E0%B8%B3%E0%B8%99%E0%B8%B1%E0%B8%81%E0%B8%9A%E0%B8%A3%E0%B8%B4%E0%B8%AB%E0%B8%B2%E0%B8%A3%E0%B9%81%E0%B8%A3%E0%B8%87%E0%B8%87%E0%B8%B2%E0%B8%99,%E0%B9%81%E0%B8%A5%E0%B8%B0%E0%B9%80%E0%B8%9E%E0%B8%A8%E0%B8%AB%E0%B8%8D%E0%B8%B4%E0%B8%87%201%2C429%2C709%20%E0%B8%84%E0%B8%99, Accessed 12 May 2024.

[24] ILO. (2022). Home truths: Access to adequate housing for migrant workers in the ASEAN region. Retrieved from: https://www.ilo.org/publications/home-truths-access-adequate-housing-migrant-workers-asean-region, Accessed 12 May 2024.

[25] Thongpan S. Problems and policy recommendations related to Transnational Migrant Workers in Thailand: the result of Research Synthesis supported by the National Research Council of Thailand [in Thai]. J Multidisciplinary Acad Res Dev. 2020;2(4):1–20.

[26] Social Security Office. (2022). Social Security Scheme Rule and Law. Retrieved from: https://www.sso.go.th/wpr/main. Accessed 1 July 2024.

[27] Office of Permanent Secretary. (2022). Policies and operating guidelines for health examinations and health insurance for foreign workers [in Thai]. Retrieved from: https://www.uckkpho.com/wp-content/uploads/2023/01/Presentation_%E0%B8%81%E0%B8%A8%E0%B8%A0.pdf Accessed 1 July 2024.

[28] Ruksanong S. (2015). Problem of employees’ welfare [in Thai]. Retrieved from: http://law.master.kbu.ac.th/StudentTheses/2558/2558-012.pdf. Accessed on 14 June 2024.

[29] Harkins B. (2019). Thailand Migration Report 2019: United Nations Thematic Working Group on Migration in Thailand. Retrieved from: https://thailand.un.org/sites/default/files/2020-06/Thailand-Migration-Report-2019.pdf. Accessed on 18 June 2024.

[30] Khai TS. Socio-ecological barriers to access COVID-19 vaccination among Burmese irregular migrant workers in Thailand. J Migration Health. 2023. 10.1016/j.jmh.2023.100194. 8. 37396687 10.1016/j.jmh.2023.100194 PMC10292913

[31] Division of Health Economic and Health Security. (2024). Policy and guidelines for health check and security for migrant worker [in Thai]. Retrieved from: https://www.uckkpho.com/wp-content/uploads/2023/01/Presentation_%E0%B8%81%E0%B8%A8%E0%B8%A0.pdf, Accessed 10 May 2024.

[32] Gushulak BD, Weekers J, MacPherson DW. Migrants and emerging public health issues in a globalized world: threats, risks and challenges, an evidence-based framework. Emerg Health Threats J. 2009;2(10):1–12. 10.3134/ehtj.09.010. 10.3134/ehtj.09.010 PMC3167650 22460280

[33] Daynes L. The health impacts of the refugee crisis: a medical charity perspective. Clin Med. 2016;16(5):437–40. 10.7861/clinmedicine.16-5-437. 10.7861/clinmedicine.16-5-437 PMC6297302 27697805

[34] World Health Organization. (2022). Migrant and Health: Key Issues. Retrieved from: https://www.euro.who.int/__data/assets/pdf_file/0005/293270/Migration-Health-Key-Issues-.pdf. Accessed 14 September 2022.

[35] Norredam M, Krasnik A, Moller Sorensen T, Keiding N, Joost Michaelsen J, Sonne Nielsen A. Emergency room utilization in Copenhagen: a comparison of immigrant groups and Danish-born residents. Scandinavian Journal of Public Health, 2004;32(1):53–59. 10.1080/14034940310001668. PMID: 15068339. 10.1080/14034940310001659 14757549

[36] Barnett ED, Walker PF. Role of immigrants and migrants in Emerging Infectious diseases. Med Clin N Am. 2008;92:1447–58. 10.1016/j.mcna.2008.07.001. 19061761 10.1016/j.mcna.2008.07.001 PMC7094553

[37] Rechel B, Mladovsky P, Ingleby D, Mackenbach JP, McKee M. Migration and health in an increasingly diverse Europe. Lancet. 2013;381(9873):1235–45. 10.1016/S0140-6736(12)62086-8. 23541058 10.1016/S0140-6736(12)62086-8

[38] Montiel I, Park J, Husted BW, Velez-Calle A. (2022). Tracing the connections between international business and communicable diseases. Journal of International Business Studies. Retrieved from: https://link.springer.com/article/10.1057/s41267-022-00512-y. Accessed 19 September 2022. 10.1057/s41267-022-00512-y PMC8942389 35345569

[39] Kunnu W, Pasunon P. The affecting of Migrant Labour in Surat Thani Municipality, Thailand [in Thai]. J Humanit Social Sci Thaksin Univ. 2015;10(1):75–94.

[40] Barwick H. (1992). The Impact of Economic and Social Factors on Health (Online). https://www.moh.govt.nz/notebook/nbbooks.nsf/0/7406A2222432E2914C2565D7000E5791/$file/The%20Impact%20of%20economic%20and%20social%20factors%20on%20health.pdf, 14 September 2022.

[41] World Health Organization. (2011). Taking sex and gender into account n emerging infectious disease programmes: An analytical framework. Retrieved from: https://iris.who.int/bitstream/handle/10665/207693/9789290615323_eng.pdf?sequence=1. Accessed 14 September 2022.

[42] Al-Worafi YM. Infectious Disease causes and Risk factors in developing countries: adults. In: Al-Worafi YM, editor. Handbook of Medical and Health Sciences in developing countries. Cham: Springer; 2024. 10.1007/978-3-030-74786-2_328-1.

[43] Samsudin EZ, Yasin SM, Ruslan N, Abdullah NN, Noor AAF, Hair AFA. Socioeconomic impacts of airborne and droplet-borne infectious diseases on industries: a systematic review. BMC Infect Dis. 2024;24:93:1–37. 10.1186/s12879-024-08993-y. 38229063 10.1186/s12879-024-08993-y PMC10792877

[44] Answer MK, Islan T, Khan MA, Zaman K, Nassani AA, Askar SE, Abro MMZ, Kabbani A. (2020). Identifying the Potential Causes, Consequences, and Prevention of Communicable Diseases (Including COVID-19). Biomed Research International, 2020: 1–12. 10.1155/2020/8894006. 10.1155/2020/8894006 PMC7643374 33204725

[45] Zhang XX, Jin YZ, Lu YH, Huang LL, Wu CX, Lv S, Chen S, Chen Z, Xiang H, Zhou XN. Infectious disease control: from health security strengthening to health systems improvement at global level. Global Health Res Policy. 2023;38:1–8. 10.1186/s41256-023-00319-w. 10.1186/s41256-023-00319-w PMC10478312 37670331

[46] Marou V, Vardavas CI, Aslanoglou K, Nikitara K, Plyta Z, Leonardi-Bee J, Atkins K, Condell O, Lamb F, Suk JE. The impact of conflict on infectious disease: a systematic literature review. Confl Health. 2024;18:27:1–32. 10.1186/s13031-023-00568-z. 38584269 10.1186/s13031-023-00568-z PMC11000310

[47] Suhrcke M, Stuckler D, Suk JE, Desai M, Senek M, McKee M, Tsolova S, Basu S, Abubakar I, Hunter P, Rechel B, Semenza JC. The impact of economic crises on Communicable Disease Transmission and Control: a systematic review of the evidence. PLoS ONE. 2011;6(6):1–12. 10.1371/journal.pone.0020724. 10.1371/journal.pone.0020724 PMC3112201 21695209

[48] Gori L, Mammana C, Manfredi P, Michetti E. Economic development with deadly communicable diseases and public prevention. J Public Econ Theor. 2021;24(5):912–43. 10.1111/jpet.12560. 10.1111/jpet.12560

[49] Center for Disease Control and Prevention. (2022). Melioidosis. Retrieved from: https://www.cdc.gov/melioidosis/index.html#:~:text=Melioidosis%2C%20also%20called%20Whitmore’s%20disease,in%20contaminated%20soil%20and%20water. Accessed 10 April 2023.

[50] Tipayamongkholgul M, Podang J, Siri S. Spatial Analysis of Social Determinants for Tuberculosis in Thailand. J Med Assoc Thai. 2013;96(Suppl5):S116–21. 24851581

[51] Zebua HI, Jaya IGNM. Spatial autoregressive to model tuberculosis cases in Central Java Province in 2019. Jurnal Matematika Murni Dan Aplikasi. 2022;7(2):240–8. 10.18860/ca.v7i2.13451

[52] Chen X, Emam M, Zhang L, Rifhat R, Zhang L, Zheng Y. Analysis of spatial characteristics and geographic weighted regression of Tuberculosis prevalence in Kashgar, China. Prev Med Rep. 2023;35:102362. 10.1016/j.pmedr.2023.102362. 37584062 10.1016/j.pmedr.2023.102362 PMC10424202

[53] Barankanira E, Molinari N, Niyongabo T. Spatial analysis of HIV infection and associated individual characteristics in Burundi: indications for effective prevention. BMC Public Health. 2015;16(118). 10.1186/s12889-016-2760-3. 10.1186/s12889-016-2760-3 PMC4743168 26847711

[54] Qin Q, Guo W, Tang W, Mahapatra T, Wang L, Zhang N, Ding Z, Cai C, Cui Y, Sun J. Spatial analysis of the human immunodeficiency Virus Epidemic among men who have sex with men in China, 2006–2015. Clin Infect Dis. 2017;64(7):956–63. 10.1093/cid/cix031. 28362948 10.1093/cid/cix031 PMC5439342

[55] Rashid M, Chand S. Socio-economic factors of misconception about HIV/AIDS among ever-married women in Punjab: a comparison of non-spatial and spatial hierarchical Bayesian Poisson model. Kuwait J Sci. 2019;46(4):33–46.

[56] Ispriyanti D, Prahutama A, Taryono APN. Modelling Space of Spread Dengue Hemorrhagic Fever (DHF) in Central Java use Spatial Durbin Model. Journal of Physics: Conference Series 2018;1025:012–112.

[57] Jaisankar R, Kesavan J, Varadha RS. Spatial Autoregressive Model for Predicting the Dengue incidences in Tamil Nadu, India. Gedrag Organisatie Rev. 2020;33(3):178–87. 10.37896/GOR33.03/418. 10.37896/GOR33.03/418

[58] Sabil RM, Sastri R. On the modelling of Leprosy Prevalence in South Sulawesi using spatial Autoregressive Model. Indonesian J Stat Its Appl. 2020;4(2):245–53. 10.29244/ijsa.v4i2.529

[59] Amiri L, Torabi M, Deardon R, Pickles M. Spatial modeling of individual-level infectious disease transmission: tuberculosis data in Manitoba, Canada. Stat Med. 2021;40(7):1678–704. 10.1002/sim.8863. 33469942 10.1002/sim.8863

[60] Shrestha N. Detecting Multicollinearity in Regression Analysis. Am J Appl Math Stat. 2020;8(2):39–42. 10.12691/ajams-8-2-1. 10.12691/ajams-8-2-1

[61] Figueroa-Munoz J, Ramon-Pardo P. Tuberculosis control in vulnerable groups. Bull World Health Organ. 2008;86(9):733–5. 10.2471/BLT.06.038737. 18797650 10.2471/BLT.06.038737 PMC2649499

[62] Pavli A, Maltezou H. Health problems of newly arrived migrants and refugees in Europe. J Travel Med. 2017;24(4):1–8. 10.1093/jtm/tax016. 10.1093/jtm/tax016 28426115

[63] Meaza A, Tola HH, Eshetu K, Mindaye T, Medhin G, Gumi B. Tuberculosis among refugees and migrant populations: systematic review. PLoS ONE. 2022;17(6):1–11. 10.1371/journal.pone.0268696. 10.1371/journal.pone.0268696 PMC9182295 35679258

[64] Department of Disease Control. (2021). Guidelines for screening for hepatitis B and C infection in the target population at risk to forward into the treatment system for local government organizations [in Thai]. Retrieved from: http://utoapp.moph.go.th/e_doc/views/uploads/6270a15361f68-7d09d7ae39f638ffa6a8b0f37fe78f35-1288.pdf. Accessed 17 February 2023.

